# Is Single-stage Revision Safe Following Infected Total Knee Arthroplasty? A Critical Review

**DOI:** 10.7759/cureus.1629

**Published:** 2017-08-30

**Authors:** Raju Vaishya, Amit Kumar Agarwal, Sudheer K Rawat, Harsh Singh, Vipul Vijay

**Affiliations:** 1 Department of Orthopedics, Indraprastha Apollo Hospital, New Delhi

**Keywords:** periprosthetic infection, infected total knee arthroplasty, single stage revision, two stage revision

## Abstract

With the improvement in outcomes and modern prosthesis design, total knee arthroplasty (TKA) has now become a commonly performed surgery. It is postulated that a total of 2-5% of the primary and revision TKA becomes infected every year, requiring a revision procedure which to date is the conventional two-stage revision. The diagnosis and treatment of these periprosthetic infections is a major and challenging task, as it requires precise identification of the pathogen, meticulous debridement, and postoperative rehabilitation. To date, there have been very few studies in existing literature comparing the outcomes of single-stage versus two-stage procedure in infected TKA. The aim of the review was to provide the clinicians an insight into the outcome of the single-stage procedure compared to two-stage procedures and to suggest ways to improve the results further. In the following critical review, a total of 669 cases that underwent either a single or two-stage revision for infected TKA were studied. The postoperative functional scores were comparable in most studies during the early postoperative period. Our data supports the use of a single-stage revision surgery in infected TKA as an alternative to a conventional two-stage procedure. However, larger prospective and multicentric trials are required to validate our ﬁndings.

## Introduction and background

With improved surgical outcomes and modern prosthesis designs, total knee arthroplasty (TKA) has become a popular surgery. It is postulated that a total of 2-5% of the primary and revision TKAs becomes infected every year [1-4], requiring a revision procedure, which to date is the conventional two-stage revision.

The diagnosis and treatment of these periprosthetic infections is a major and challenging task, as it requires precise identification of the pathogen, an antibiotic strategy, meticulous debridement, careful surgical technique, and postoperative rehabilitation. Nevertheless, this in itself is a cumbersome and time-consuming process. The economic burden of the patient has to be taken into consideration, and the prolonged time to recovery is another big challenge that the orthopedic surgeons face [5]. Some of the other major challenges posed in the management of these infected TKA cases include: the infecting organisms' virulence, type and number of isolates, systemic involvement, the chronicity of illness, soft tissue involvement, and the condition of the available bone stock.

There is a need for this critical review, as to date there are very few studies in existing literature comparing the outcomes of single-stage versus two-stage procedure in infected TKA. The rate of success of two-stage revision has been reported to be between 72% and 100% in eradicating infection and re-establishing a functioning joint [6-11]. The conventional two-stage procedure has proved to be very effective to date, but carries morbidity due to prolonged period of treatment as well as economic overburden. Single-stage revision procedure, though in its nascent stages, has proved to be highly efficacious in selected cases.

This review aims to further study this in detail to provide clinicians an insight into the outcome of the single-stage procedure and suggest ways to improve the results further.

## Review

Two-stage revision TKA

**Figure 1:**
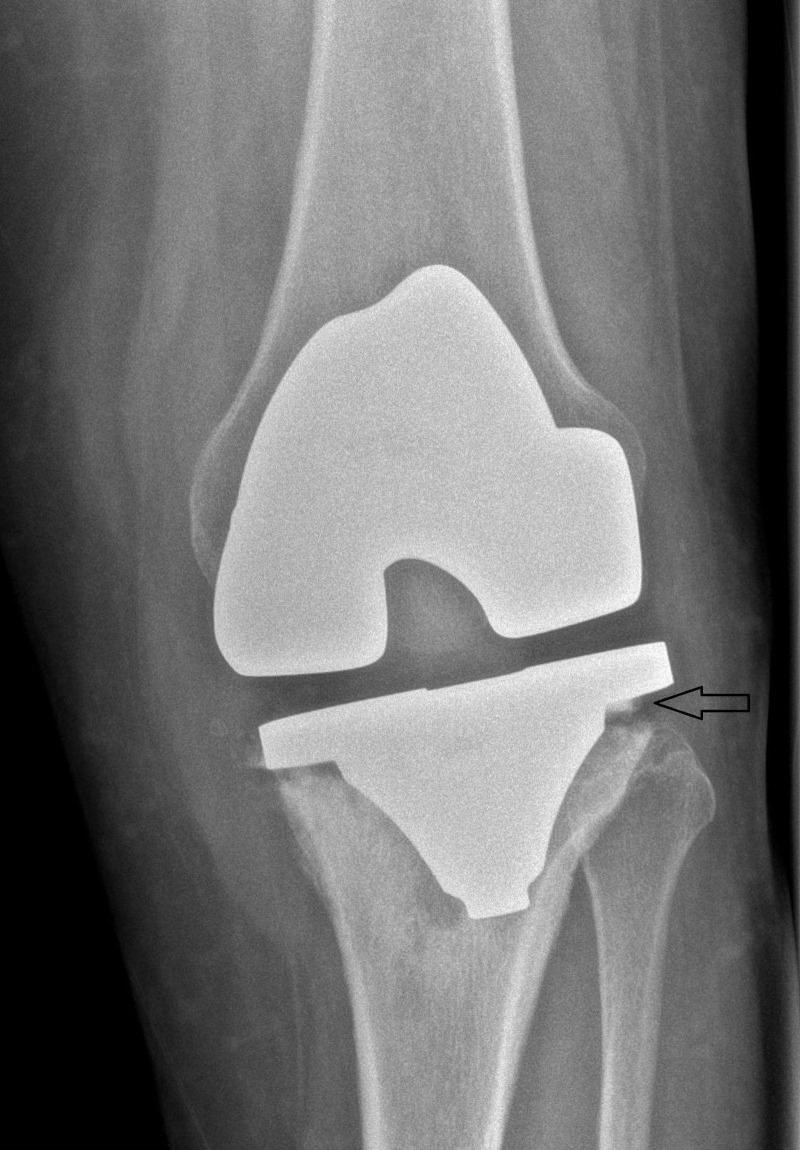
Preoperative X-ray of the Left Knee (Anteroposterior View) Showing Loosening of Tibial Component

**Figure 2:**
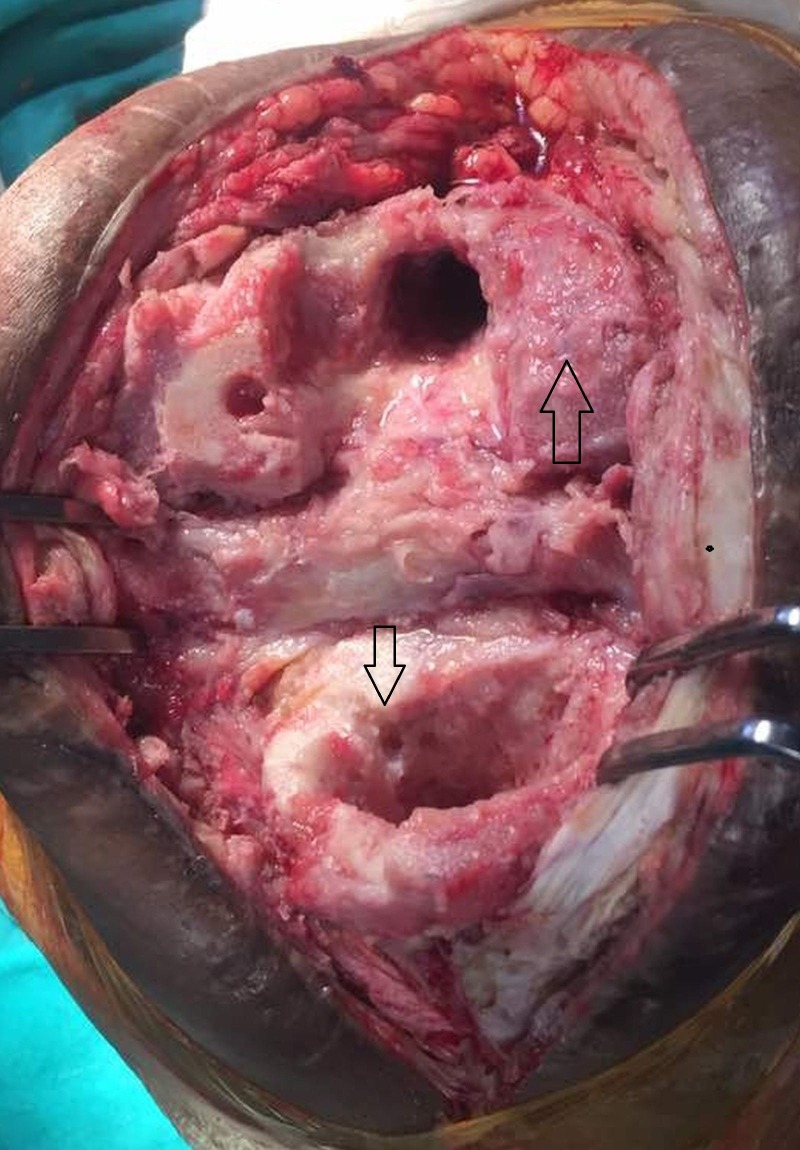
Intraoperative Picture After Radical Debridement and Removal of Tibial and Femoral Implants

**Figure 3:**
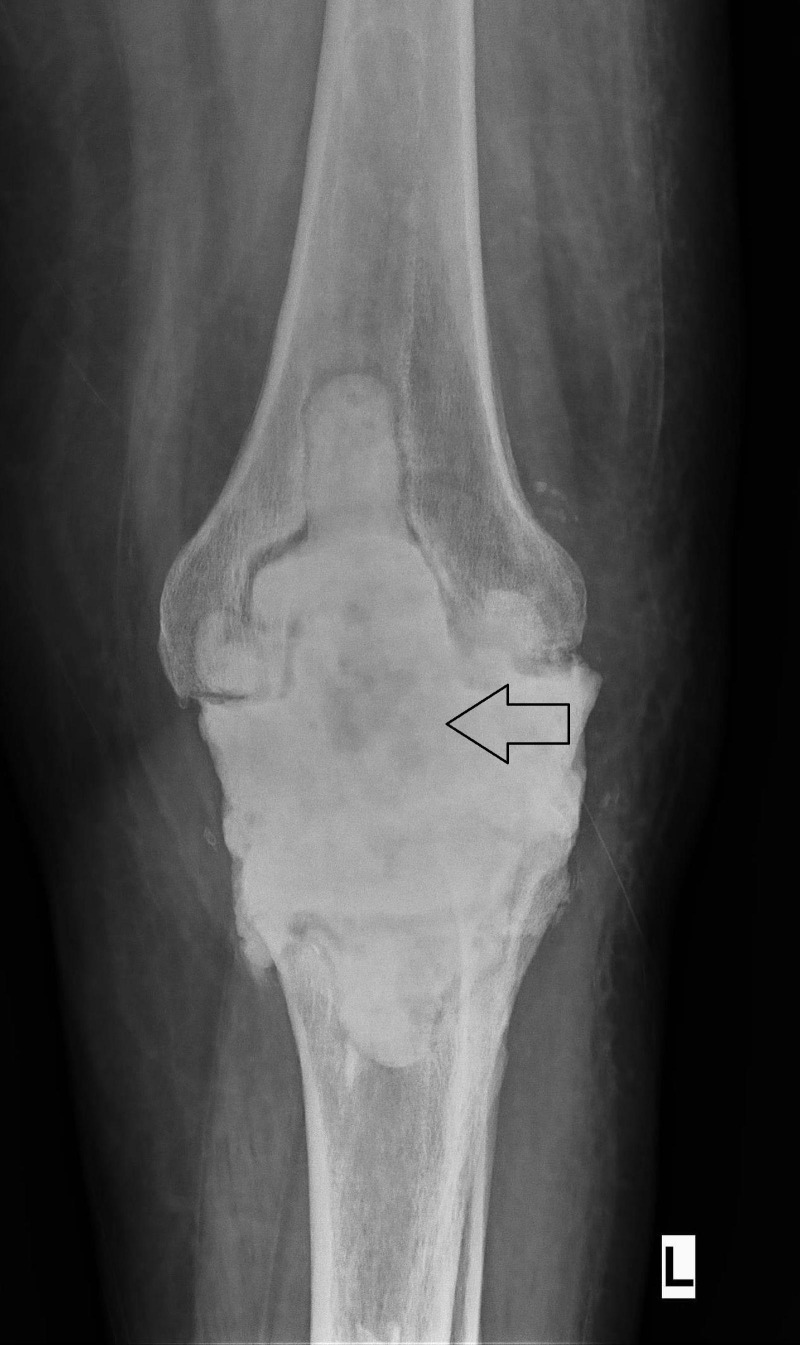
X-ray of the Left Knee Showing the Antibiotic-impregnated Bone Cement as Spacer after the Removal of Infected Implants

A two-stage method is the most common technique used for revision TKA worldwide. It involves two steps: the first step is the removal of the infected prosthesis and radical debridement. The resultant gap is filled with an antibiotics-containing cement spacer to maintain the muscle and soft-tissue tension in the knee joint. Articulating spacers are used for few degrees of movement at the knee joint. The second step involves reimplantation after the removal of the antibiotic-impregnated cement spacer at the interval of six to eight weeks (Figures 1-3).

**Figure 4:**
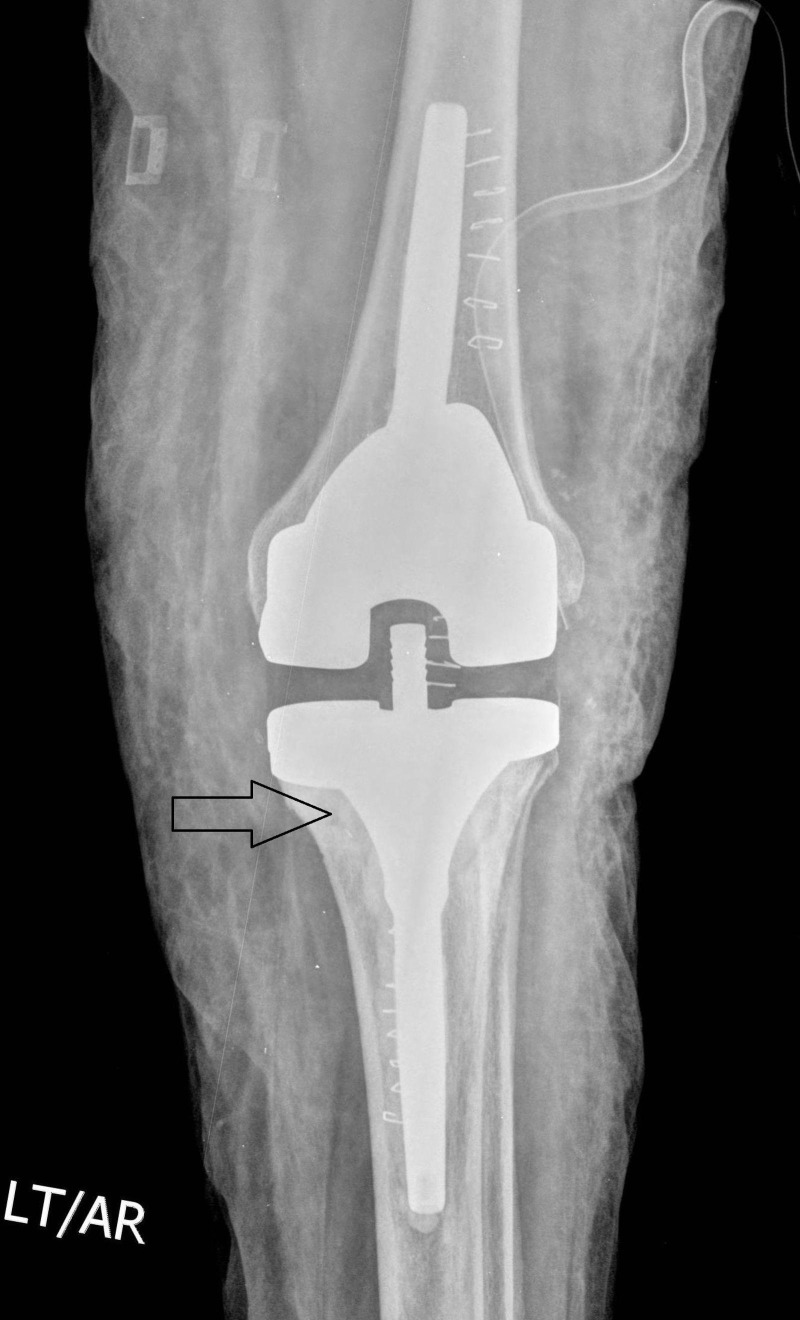
Postoperative X-ray (Anteroposterior View) Showing Revision Total Knee Arthroplasty (TKA)

**Figure 5:**
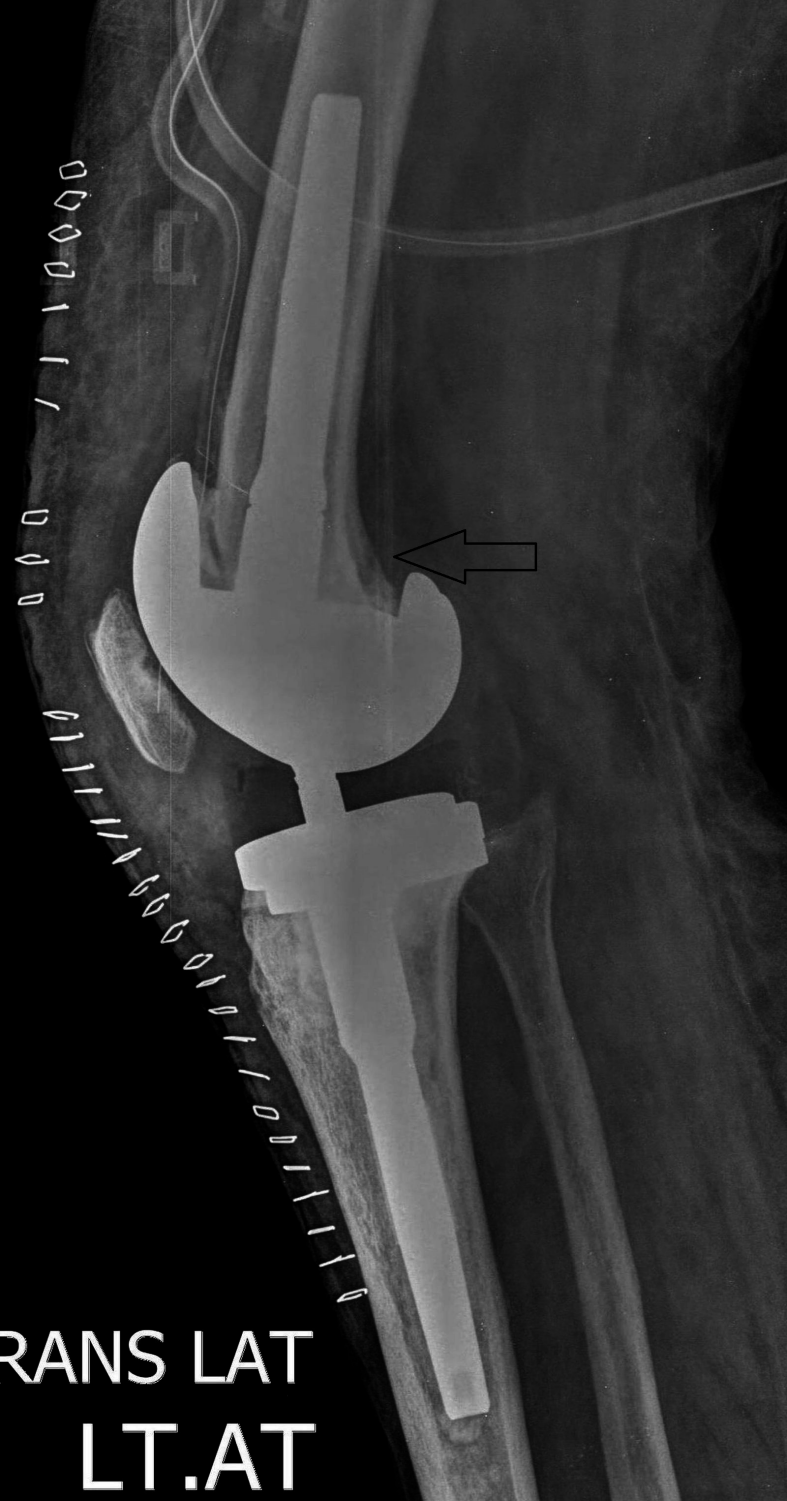
Postoperative Lateral View of the Knee Showing Revised Total Knee Arthroplasty (TKA)

Parenteral antibiotics are adminstered according to culture sensitivity. The inflammatory markers are monitored until they come down to a normal range. After around six to eight weeks, the definitive TKA procedure is performed (Figures 4-5).

During the single-stage procedure, the revision of the prosthesis is conducted at the same stage after removal of the infected implant. Nowadays, the popularity of the ‘one-stage’ procedure is increasing due to the disadvantages of the two-stage revision (Table 1).

**Table 1:** Comparison of Single vs. Two-stage Revision of Infected Total Knee Arthroplasty (TKA)

	Two-stage revision	Single-stage revision
1	Two major consecutive surgeries are needed	Single surgery
2	Extended hospital stay leads to more comorbidities	Less comorbidity
3	Due to the long interval between two-stage procedure muscles get weak and soft tissue gets contraction	Improved functional knee outcome and no risk of soft tissue contraction or muscle weakness
4	Financial burden due to an extended hospital stay and antibiotics medication	Less financial burden due to single surgery and less duration of hospitalization

Tibrewal, et al. [12], in their cohort of 50 patients with a mean follow-up of 10.5 years, had identified only one true recurrent infection confirmed with a positive microbiological result at the further revision procedure, representing a 98% success rate. They believed that extensive debridement, identification of the infecting organisms, and proper antibiotic treatment, as directed in discussion with a microbiologist, are the key factors in the success of single-stage revision for the management of an infected TKA. It should gain further acceptance, and they agree with a recent review that advocates its use in selected cases.

In a review by Haddad, et al. [13], out of a total of 102 cases, 28 cases with single-stage revision and 74 cases with two-stage revision were included. The mean follow-up duration of the study was 6.5 years. In the single-stage revision group no reinfection was seen, whereas in two-stage revision, ﬁve patients (7%) developed recurrence of the infection. These patients underwent a further two-stage revision TKA and had their infection controlled at the last follow-up. The Knee Society Score (KSS) was higher in the single-stage group at the two-year follow-up compared to the two-stage group. There was no evidence of loosening of the prosthesis radiologically at the last follow-up in either group.

In a study by Baker, et al. [14], Patient Reported Outcome Measures (PROMs) for 33 single-stage and 89 two-stage revisions were analyzed in combination with data from the National Joint Registry of England and Wales. Outcomes were measured by various knee scores. No significant statistical difference was found between both groups for any reported outcome measure. The mean Oxford Knee Score (OKS) following surgery was 22.8 for two-stage and 24.9 for single-stage, which is better compared to two-stage revision TKA. The mean EuroQol five-dimension questionnaire (EQ-5D) index following surgery was 0.495 for single and 0.473 for two-stage. Patients reporting Excellent/Very good/Good satisfaction levels were similar in both the groups. In total, 66% single and 60% two-stage operations were rated 'successful'. This study did not find any demonstrable beneﬁt of one technique over the other using a variety of PROMs. It did not discuss the rate of reinfection, and a third parameter for the study was subjective. There was a marginal benefit in all the three parameters used in the study of single-stage compared to two-stage revision TKA.

Klatte, et al. [15] in their study between 2001-11 suggested that fungal periprosthetic joint infections are rare but can lead to chronic complications. They evaluated the results of their single-stage revision technique. A total of 14 patients were treated for a periprosthetic fungal infection. Unfortunately, two patients died of unrelated causes. Out of four cases of TKA, one patient had delayed wound healing and subsequently underwent exploration without revision of the components. There was no sign of infection. This patient suffered a periprosthetic femoral fracture 29 months postoperatively. At this revision, no bacterial or fungal growth was detected. In another patient with a TKA, further infection with *Candida parapsilosis* required revision with a second single-stage two months after the surgery. This patient, who is immunocompromised as a result of steroid use for more than 15 years for chronic obstructive lung disease, also has diabetes mellitus and developed recurrent necrosis of the skin, requiring soft-tissue reconstruction on two occasions. One year later, he presented with a sinus around the knee, and on aspiration, intraoperative samples showed growth of *Staphylococcus epidermidis*. A third single-stage exchange revision was undertaken, and there have been no further signs of infection for more than 1.7 years. Thus, at a mean follow-up of seven years, there has been one further infection. A single-stage revision following fungal periprosthetic infection is feasible, with an acceptable rate of a satisfactory outcome.

Gulhane, et al. [16] in a review reported a 97% success rate for single-stage revision performed at their institute, after a minimum follow-up of two years. They used a technique similar to the one used by Tibrewal, et al. with cement loaded with vancomycin and tobramycin, and allograft. However, their study did not mention any specific sample size, and not much information was included regarding the functional recovery or cost analysis. The gold standard procedure in their institution is a two-stage revision. They concluded that single-stage revision is a preferred option and can be used judiciously for appropriate patients.

Single-stage revision TKA

Mortazavi, et al. [17] in their retrospective cohort study of 117 cases stated that the mean follow-up period was 3.8 years. The rate of reinfection was 28%. The common organism isolated was *Staphylococcus aureus*, requiring revision surgery. The success rate of single-stage revision TKA was 72%. It needs longer follow-up to validate its result.

Von Foster, et al. [18] in their study had 118 cases who underwent single-stage revision TKA using specific antibiotic-loaded cement. This study had a success rate of 73%, similar to Mortazavi, et al. It had a long follow-up, averaging 10.5 years. Seventy-six cases were cured as a result of this single-stage revision TKA. The reinfection rate was 27%, which later on went to revision by the two-stage procedure. Of these, 20 cases of one-stage revision TKA failed in the treatment of periprosthetic infections after TKA.

In a case series of 12 cases of revision TKA by Parkinson, et al. [19], there were zero rates of reinfection after single-stage revision of infected TKA, with a success rate of 100%. However, it had only 12 cases, which was a very small group, and the follow-up was conducted after a short period of only two years. In order to consider its results, long follow-up is needed. Postoperative knee functional score was not measured in this study. While knowing the reinfection rate at the final outcome is indeed necessary, knowing the functional knee score as well is very important.

Buechel, et al. [20] had a case series of 22 patients of infected TKAs treated by a single-stage revision. Though the number was small, it had a long follow-up period of mean 10.2 years. Of these, 90.9% were free of recurrent infection. The rate of reinfection was 9.1%, which is similar to other studies of single-stage revision. They performed radical debridement with normal saline and betadine irrigation. Their outcome was based on two parameters: reinfection and KSS. Knee scores averaged 79.5, with 85.7% good or excellent results.

Singer, et al. [21] retrospectively reviewed a prospective study of 63 cases of single-stage revisions between 2004-6. All cases were treated with microorganism-specific antibiotics locally and systemically, excluding patients with methicillin-resistant *Staphylococcus aureus* (MRSA),methicillin-resistant*Staphylococcus epidermidis* (MRSE)*,* or unknown microorganisms. The patients' follow-ups were conducted every three months where the OKS and KSS were assessed. The minimum follow-up was 24 months. The chances of infection control were influenced by the duration of infection. The mean KSS at two years after surgery was 72 points, the mean Knee Society function score was 71 points, and the mean Oxford knee score was 27 points with a success rate of 95% and a reinfection rate of 5%. However, this indicates that follow-up was of a three-year duration, which is a short follow-up period. This result was based on reinfection and postoperative knee functional score.

In the following critical review, a total of 669 cases were studied, which underwent either a single or two-stage revision for infected TKA (Table 2).

**Table 2:** Critical Review of the Study NR - not recorded

Sr.no	Name of study	Study type	No. Of cases	Follow-up (mean) yrs	Commonly isolated organism	Reinfection rate	Antibiotics used in cement	Revision after one stage	Success rate %
1	Tibrewal, et al. [12] (2014)	Case series	50	10.5	Staphylococcus Aureus	8%	Cement + sensitive antibiotic	2%	98%
2	Haddad, et al. [13] (2014)	Cohort	102 1st 28 2nd 74	6.5	Coagulase negative Staphylococcus Aureus (33%)	1st-0% 2nd-7%	1 gm vancomycin and 1 gm gentamycin in 40 gm of Placos	-	100%
3	Baker, et al. [14] (2013)	Retro Cohort	195 1st-33 2nd-89	7mth	NR	NR	Cement + specific antibiotics	NR	66%
4	Klatte, et al. [15] (2014)	Case series	4	7	Candida Parapsilosis and Staph Epidermidis	25%	10% of admixture (vancomycin, clindamycin and gentamycin)	25%	75%
5	Gulhane, et al. [16] (2012)	Retro cohort	-	2.5	NR	-	Antibiotic inpregnated cement gentamycin + specific antibiotic systemic	-	97%
6	Mortazavi, et al. [17] (2011)	Retro cohort	117	3.8	Staph aureus (27%), Staph. epidermidis (16%), Group B Streptococci (4.6%)	28%	Antibiotic cement	28%	72%
7	von Foerster, et al. [18] (1991)	Retro cohort	104	10.5	NR	27%	Antibiotic containing cement	27%	73%
8	Buechel, et al. [20] (2004)	Case series	22	10.2	NR	10%	Specific antibiotic + cement	10%	90.9%
9	Parkinson, et al. [19] (2011)	Case series	12	2	NR	0%	Antibiotic cement	0%	100%
10	Singer, et al. [21] (2012)	Retro cohort	63	3	NR	5%	Antibiotic cement	5%	95%

Most reviews done to date have focused on validating the usefulness versus futility of the single-stage procedure. In the following review, many more parameters were incorporated and finer details were noted (Table 3).

**Table 3:** Early Postoperative Knee Scores in Various Studies OKS - Oxford Knee Score, HSS - Hospital for Special Surgery, NR - not recorded, KSS - Knee Society Score.

SR.NO	Study	Functional score	Results
1	Haddad, et al. [13]	Knee Society Score	KSS-56 points improvement in single stage group, 45 points improvement in two-stage group (at two-year follow-up)
2	Tibrewal, et al. [12]	Oxford Knee Score	20 points improvement (OKS) at one-year follow-up
3	Klatte, et al. [15]	Hospital for Special Surgery Knee Score	24 points improvement (HSS) at mean follow-up of 7 years
4	Gulhane, et al. [16]	Post operation five-year infection-free rate.	-
5	Baker, et al. [14]	Patient reported outcome measures, Oxford Knee Score	24.9 (single-stage group), 22.8 (two-stage group)-OKS
6	Mortazavi, et al. [17]	NR	NR
7	von Foerster, et al. [18]	NR	NR
8	Buechel, et al. [20]	Knee Society Score.	KSS scores averaged 79.5
9	Parkinson, et al. [19]	NR	NR
10	Singer, et al. [21]	Knee Society Score, Oxford Knee Score	KSS score was 71 points, and the OKS-12 score was 27 points

Most studies did not note any significant difference between the two procedures. The postoperative functional scores were comparable in most studies during the early postoperative period. Selection of the right case and meticulous preoperative planning, coupled with the correct operative technique can result in equally good outcomes, following a single-stage revision procedure for infected TKA. It is noted in most studies of relevance that some re-operations are directly linked to the success or failure of the procedure [22-25]. Good surgical practices such as adequate debridement, chlorohexidine packing for 30 minutes followed by re-draping, and use of a fresh set of instruments can harvest promising results [26-30]. It is recommended that a total of 10 percent of the antibiotic admixture per 40 gram of cement is used. However, some surgeons prefer to use a higher concentration of antibiotics in cement. A postoperative antibiotic period ranging from 7-12 weeks was used in most of the procedures [31-36]. After these recommendations from the preliminary data and early experience, it is concluded that a single-stage revision procedure for infected TKA can be a safe and cost-effective measure when performed in the cases with specific indication. This can also be an effective alternative to the conventional two-stage procedure. However, further studies need to be conducted regarding this concern.

The drawback of the review is that it is hard to compare between various outcome measures as different studies have used different parameters. Very few studies are available in literature, hence limiting the validity.

## Conclusions

The two-stage revision TKA procedure is considered the gold standard for the management of infected TKA. However, this systematic review demonstrates a much larger body of evidence to suggest the use of single-stage procedure over two-stage procedure. Our data supports the use of single-stage revision surgery in infected TKA cases as an alternative to a two-stage procedure, in carefully selected patients. None of the studies described here offer definitive evidence to support either technique. Multiple prospective and multicenter trials are needed to validate our ﬁndings.
